# IgG4 Autoantibodies Attenuate Systemic Lupus Erythematosus Progression by Suppressing Complement Consumption and Inflammatory Cytokine Production

**DOI:** 10.3389/fimmu.2020.01047

**Published:** 2020-06-17

**Authors:** Qingjun Pan, Haiyan Xiao, Lei Shi, Yiming He, Jun Cai, Jing Wu, Aifen Li, Lin Ye, Chen Yang, Hua-feng Liu

**Affiliations:** ^1^Key Laboratory of Prevention and Management of Chronic Kidney Disease of Zhanjiang City, Institute of Nephrology, Affiliated Hospital of Guangdong Medical University, Zhanjiang, China; ^2^Department of Cellular Biology and Anatomy, Augusta University, Augusta, GA, United States

**Keywords:** systemic lupus erythematosus, autoantibody, IgG4, complement consumption, inflammatory cytokine

## Abstract

Pathogenic autoantibodies can cause inflammation and tissue injury in systemic lupus erythematosus (SLE). Although IgG4 is considered non-inflammatory owing to the unique structure of its hinge region, the role of IgG4 autoantibodies in SLE remains largely unknown. The titers of serum anti-nuclear-IgG antibodies (ANA-IgG) and anti-nuclear-IgG4 antibodies (ANA-IgG4) in newly diagnosed SLE patients were detected. The effects of IgG4 purified from SLE patients (SLE IgG4) and healthy controls on complement consumption and inflammatory cytokine production were evaluated *in vitro*. The therapeutic effects of mouse IgG1 (functionally resembles human IgG4) purified from lupus-prone MRL-*lpr/lpr* mice (lupus IgG1) and control mice on disease progression were examined in MRL-*lpr/lpr* mice. The results showed that SLE patients with equal titers of total serum ANA-IgG (1:3,200) were divided into group I with lower ANA-IgG4 titers (≤ 1:10) and group II with higher ANA-IgG4 titers (≥ 1:100), and disease activity, inflammatory cytokine production, complement consumption, and renal-function parameters in group I SLE patients were more severe than those in group II. Further, compared with control IgG4, SLE IgG4 inhibited complement consumption by autoantibody-autoantigen immune complexes, and also inhibited inflammatory cytokines production by SLE PBMCs *in vitro*. Moreover, compared with control IgG1, lupus IgG1 exhibited a therapeutic effect on lupus by attenuating disease progression in MRL-*lpr/lpr* mice. These findings, for the first time, suggest that IgG4 autoantibodies can attenuate SLE progression by suppressing complement consumption and inflammatory cytokine production. Hence, this study may provide novel therapeutic strategies against SLE and other autoimmune diseases.

## Introduction

Systemic lupus erythematosus (SLE) is a systemic autoimmune disorder involving a wide range of autoantibodies and inflammatory cytokines (1). Autoantibodies, particularly antinuclear antibodies (ANAs), were proposed to play crucial pathogenetic roles in SLE development and may also aid in SLE diagnosis (2, 3). Autoantibody-autoantigen immune complexes (ICs) mediate most of the inflammation and tissue dysfunction associated with SLE, which occur primarily through activation of the complement system and complement abnormalities (4–6). Although the roles of the complement system in IC processing have been well-characterized in SLE (7), the abilities of different autoantibody subtypes in the promotion of complement consumption in SLE are still under investigation.

Immunoglobulin G (IgG) is a major serum immunoglobulin (representing ~ 75% of all Igs) and is divided into four subtypes: IgG1 (60–70%, 5–12 mg/mL), IgG2 (15–20%, 2–6 mg/mL), IgG3 (5–10%, 0.5–1.0 mg/mL), and IgG4 (4–6%, 0.2–1.0 mg/mL) (8, 9). In autoimmune diseases, the inflammatory responses induced by each autoreactive IgG subtype depends on its ability to activate effector functions by binding to complement proteins, Fc γ receptors, etc. IgG1 and IgG3 contribute to complement activation via binding high-affinity C1q (10). However, the IgG4 hinge region has a unique structure in terms of the amino acid sequence, arrangement, and side-chain lengths (10), which limits its ability to promote effector function by activating complement C1q (11, 12). Thus, IgG4 cannot induce a predominant immune response and is defined as a non-inflammatory molecule (13). In these regards, murine IgG1, which functionally resembles human IgG4 (14, 15), is considered an inability antibody (16), whereas the ability of IgG4 to bind the targeted antigen is the same as that of the other IgG subclasses (17, 18). In fact, natural or improved IgG4 frameworks have been used to develop therapeutic antibodies that do not require effector functions. Currently, the marketing of several IgG4-type therapeutic antibodies, including Keytruda® and OPDIVO® for PD-1 targets, has been approved (19).

Considering that a high prevalence (~25%) of the general population displays low levels of autoreactivity, represented by ANAs, it is suggested that autoantibodies serve as an important means for maintaining a healthy immune system (20–22). Hence, increasing attention has been paid to determining the clinical significance and therapeutic potential of exploiting the protective effects of autoantibodies in homeostasis (23, 24). These studies may lead to the application of natural autoantibodies as a marker for monitoring disease severity, or as a therapeutic target for autoimmune diseases (25). For instance, IgG4 autoantibodies have been universally identified in humans (26); however, their roles and possible mechanisms in autoimmune diseases, such as SLE, have not been clearly reported.

In this study, we systemically investigated the role and mechanisms of action of IgG4 autoantibodies in newly diagnosed SLE patients and lupus-prone MRL-*lpr/lpr* mice *in vitro* and *in vivo*. The results herein showed, for the first time, that IgG4 autoantibodies can attenuate SLE progression potentially via suppression of complement consumption and inflammatory cytokine production, which may provide novel therapeutic strategies against SLE and other autoimmune diseases.

## Materials and Methods

### Characteristics of SLE Patients and Healthy Controls

One hundred and six newly diagnosed, untreated SLE patients (Table 1) and 32 healthy control subjects with no significant differences in age, sex, or race were enrolled in this study from the Department of Nephrology at the Affiliated Hospital of Guangdong Medical University from January 2017 to June 2019. The patients were diagnosed according to the revised criteria of the American College of Rheumatology (ACR) for SLE (27). SLE disease activity was measured using the Systemic Lupus Erythematosus Disease Activity Index-2000 (SLEDAI-2000) score (28, 29). Patients with coinfections, allergies, other serious systemic diseases, other autoimmune disorders, or IgG4-related disease were excluded. Clinical data and laboratory results were collected. The study was approved by the ethics committee of our hospital. Written informed consent was obtained from all patients and healthy control subjects.

**Table 1:** Demographic characteristics of SLE patients and healthy control subjects.

	**Newly diagnosed SLE patients (*****n=*** **106)**			**Healthy control subjects (*n =* 32)**
	**Total SLE patients (*n =* 106)**	**Group I (*n =* 37)**	**Group II (*n =* 32)**	
Age (mean, SD)	26.3 ± 13.8	24.7 ± 11.3	25.8 ± 9.4	24.2 ± 13.6
Gender: female/male, no. (%)	92 (86.8%)/14 (13.2%)	33 (89.2%) 4 (10.8%)	27 (84.4%)/5 (15.6%)	26 (80.6%)/6 (19.4%)
Anti-dsDNA IgG-positive: no. (%)	97 (91.5%)	34 (91.9%)	30 (93.8%)	0 (0%)
Anti-nuclear IgG-positive: no. (%)	104 (98.1%)	37 (100%)	32 (100%)	0 (0%)
SLEDAI score				
Mean ± SD	15.8 ± 6.34	18.2 ± 3.97	15.9 ± 3.38	
Median (minimum, maximum)	17 (5, 29)	18 (7, 29)	15 (6, 22)	

### Mice

Female lupus-prone MRL-*lpr/lpr* mice were purchased from Shanghai SLAC Laboratory Animal Co., Ltd. (Chinese Academy of Sciences; Shanghai, China) (30) and control female C57BL/6J mice were purchased from the Guangdong Medical Laboratory Animal Center. All mice were maintained under specific pathogen-free conditions in the animal research facility at the Laboratory Animal Center of Guangdong Medical University. All experiments were performed in accordance with the guidelines of the Ethics Committee for Experimental Animals at Guangdong Medical University, which approved this study.

### Autoantibody Assay

Human and mouse serum ANA levels were primarily determinedusing a HEp-2 cell-based indirect immunofluorescence (IF) (EUROIMMUN™ Dermatology Mosaic, Euroimmun Medizinische Labordiagnostika AG, Lübeck, Germany), which serves as a gold standard for ANA determinations (31). Samples including human serum, purified human IgG4, and mouse IgG1 were serially diluted (from 1:10 to 1:10,000) for ANA testing, which was carried out following the guidelines of the manufacturer of EUROIMMUN™ Dermatology Mosaic. For human IgG4 and mouse IgG1 testing, the standard IF staining was modified using FITC-anti-human IgG4 (Abcam, Inc., Cambridge, MA, USA) or FITC-anti-mouse IgG1 (Southern Biotech Associates, Inc., Birmingham, AL, USA) as the secondary antibody, respectively.

To test the anti-nuclear IgG4 levels in humans, the ANA Euroline Profile 3 Kit (Euroimmun Medizinische Labordiagnostika AG) was employed with the modification of using alkaline phosphatase (AP)-conjugated anti-human IgG4 (Thermo Fisher Scientific, Inc., San Jose, CA USA) as the secondary antibody, and the serum samples were diluted 1:100. An indirect enzyme-linked immunosorbent assay (ELISA) method was employed to test the nuclear antigens prepared from HEp-2 cells (American Type Culture Collection, Manassas, VA, USA), which included a native protein array with hundreds of antigens (31). The nuclear antigens were coated on 96-well microplates at the optimal concentration (50 μg/mL) in coating buffer (0.58M carbonate–bicarbonate buffer, pH 9.5), with horseradish peroxidase (HRP)-conjugated anti-human IgG (AbD Serotec, Oxford, United Kingdom) as the secondary antibody. A chromogenic substrate (3,3′,5,5′-tetramethylbenzidine, [TMB]; Sigma-Aldrich, Missouri, USA) was added, and the absorbance was measured at 450 nm. The serum samples were diluted 1:100 before testing for ANA.

In addition, serum levels of anti-nuclear IgG in mice were also measured using ELISA kits. To test the anti-nuclear IgG1 and IgG3 levels in mice, an ANA Screen ELISA Kit (Zeus Scientific, Inc., Branchburg, NJ, USA) was employed with the modification of using HRP-conjugated anti-mouse IgG1, or HRP-conjugated anti-mouse IgG3 as the secondary antibody, respectively, and the serum samples were diluted 1:150. In addition, to test for anti-double-stranded DNA (dsDNA) IgG3 in mouse, an anti-dsDNA IgG ELISA Kit (Fuchun Kexin Biotech, Shanghai, China) was employed with the modification of using HRP-conjugated anti-mouse IgG3 as the secondary antibody, and the serum samples were diluted 1:150.

All samples including serum, urine, and cell culture supernatants were collected and stored at −80°C until diagnostic testing was performed.

### Serum and Urine Assays

Human serum C3 and C4 were detected by rate nephelometry using a Beckman IMMAGE 800 analyzer (Beckman-Coulter, Carlsbad, USA). Serum creatinine, blood urea nitrogen (BUN), 24-h proteinuria, urine protein, and creatinine levels were measured with a Cobas 8000 automatic biochemistry analyser (Roche Diagnostics, GmbH, Mannheim, Germany), and 24-h urinary protein was measured with an Olympus AU2700 automatic biochemistry analyzer (Olympus Corporation, Tokyo, Japan) (32). Human serum complement factor B and factor H were quantified using commercially available ELISA kits (Abcam, Inc., Cambridge, MA, USA). C3a and C5a were quantified with commercial ELISA kits (Cusabio Biotechnology Co., Ltd, Wuhan, China). The levels of interferon-γ (INF-γ), interleukin-6 (IL-6), and IL-17 in human and mouse sera, and cell culture supernatants, were also quantified using commercial ELISA kits (Life Technologies Corporation, Grand Island, NY, USA).

Twenty-four-hour urine samples were collected from mice in metabolic cages and assessed for creatinine, C3 (ADI, San Antonio, TX, USA), BUN (StressMarq Biosciences, Inc., Victoria, BC, Canada), and urinary proteins (Bio-Rad Protein Assay, Bio-Rad Laboratories, Hercules, CA, USA) levels in mice. Mouse serum complement factor B and factor H were quantified using commercially available ELISA kits (LifeSpan BioSciences, Inc., WA, USA) according to manufacturer's instructions.

### Tissue Assays

IF staining analysis was employed to measure immunoglobulin depositions and complement proteins in frozen mouse kidney sections (33). Rat anti-mouse IgG, IgG1, IgG3-FITC (Southern Biotechnology Associates, Inc., Birmingham, AL, USA), FITC-anti-mouse complement component C1q, and FITC-anti-mouse complement component C3 (Cedarlane Laboratories, Ltd., Burlington, ON, Canada) were employed. The IF staining intensity of glomeruli was scored on a scale from 0 to 4 in increments of 0.5, and the staining intensity was analyzed under an laser-scanning confocal microscope (TCS SP5 II, Leica Microsystems, Mannheim, Germany) (34).

For pathological evaluation of lupus nephritis, the kidneys were harvested at 19 weeks of age, fixed with 10% formalin, and embedded in paraffin for processing into 2-μm tissue sections. The paraffin sections were then prepared and stained with hematoxylin & eosin (H&E), periodic acid Schiff's reaction (PAS), and Masson's trichrome stain for histochemical study. Images were captured using a bright-field slide scanner (Olympus BX60) and processed using Leica software (Leica Microsystems).

### Complement Consumption Experiment Assay *in vitro*

IgG4 was isolated from the sera of high IgG4-ANA-positive SLE patients (*n* = 12; titer ≥ 1:100) or healthy control subjects (*n* = 12) by immunoaffinity column chromatography using mouse anti-human IgG4 (Thermo Fisher Scientific, Inc., San Jose, CA USA) coupled to CNBr-activated Sepharose™ 4B (GE Healthcare, Chicago, USA) and quantified using the Pierce™ BCA Protein Assay Kit (Thermo Fisher Scientific, Inc., San Jose, CA USA). Briefly, the serum was firstly diluted with three times of PBS buffer (10 mM, pH7.4), and then centrifuged (5 min, 10,000g, 4°C) and filtered through a membrane filter (0.45 μm pore size). Followed, the diluted serum was loaded onto the column with three round repeat (0.8 ml per min). Then, the column was washed with ten times of the bed volume with PBS buffer (10 mM, pH 7.4). Finally, the column was washed with ten times of the bed volume with elution buffer (0.15 M, pH 2.5, Glycine-HCl buffer), and then the flow-through fractions (1.0 ml per EP tube) were immediately neutralized to pH 7.2–7.4 with NaHCO_3_ solution (1.0 M, pH 9.0). The homogeneity and reactivity of IgG4 antibody mixtures were analyzed by reducing (dithiothreitol, DTT) and non-reducing sodium dodecyl sulfate-polyacrylamide gel electrophoresis (SDS-PAGE), immunofluorescence (IF) staining with HEp-2 cells, and analysis with the ANA Euroline Profile 3 Kit.

To investigate the influence of IgG4 autoantibodies on complement consumption, a cell-based ELISA technique was used to quantify complement system activation by measuring complement C3, C4, factor B, and factor H in the supernatant, as well as cell-surface deposition of C5b-9 (35, 36). First, HEp-2 cells were grown to 80% confluency in Dulbecco's modified Eagle's medium/nutrient mixture F-12 (DMEM/F-12; Invitrogen Corporation, Carlsbad, CA, USA) supplemented with 10% fetal bovine serum (FBS; Gibco, Life Technologies Corp., USA) at 37°C and 5% CO_2_ in a humidified incubator (Thermo Scientific, Waltham, MA, USA). Subsequently, the cells were fixed with 100% methanol for 12 min and with acetone for 1 min at −20 °C (37). The cells were then washed twice with phosphate-buffered saline (PBS; 20 mM, pH 7.4). Second, sera from ANA-IgG-positive SLE patients (*n* = 12; titer 1:3,200) were diluted 1:200 with PBS containing 0.1% bovine serum albumin (BSA) and mixed with different concentrations of IgG4. Next, the mixtures from the second step were combined with HEp-2 cells from the first step and co-cultured for 30 min at 37°C. Finally, the supernatant was collected to detect complement proteins, and the plates were used to measure C5b-9 deposition on HEp-2 cells via indirect ELISA, with the absorbance measured at 450 nm, followed by addition of a chromogenic reaction (TMB; Sigma-Aldrich, Missouri, USA), using a rabbit anti-human C5b-9 primary antibody (Abcam Inc., Cambridge, MA, USA) and HRP-conjugated goat anti-rabbit IgG (Boshide, Wuhan, China) as the secondary antibody.

### Detection of Inflammatory Cytokine Production by SLE PBMCs *in vitro*

Peripheral blood mononuclear cells (PBMCs) were obtained from the whole blood of highly ANA-IgG-positive SLE patients (*n* = 12; titer 1:3,200; the same patients to IgG4 isolated from whose sera). The influence of IgG4 autoantibodies on inflammatory cytokine production by SLE PBMCs was investigated as follows. First, nuclear antigens were prepared from the HEp-2 cells using the NE-PER^TM^ Nuclear and Cytoplasmic Extraction Reagents Kit (Thermo Fisher Scientific, Inc., Waltham, MA, USA), according to the manufacturer's instructions. The quality and reactivity of nuclear antigen preparations were analyzed by SDS-PAGE and ELISA. Second, PBMCs (1 × 10^6^ cells/well) were grown in DMEM/F12 supplemented with 25% serum of highly ANA-IgG-positive SLE patients at 37°C and 5% CO_2_ in a humidified incubator, mixed with different concentrations of IgG4 isolated from SLE patients and healthy control subjects. Third, nuclear antigens were at a final concentration of 5.0 μg/mL. Subsequently, the cells were co-cultured for 48 h. Finally, the supernatants were collected to detect the levels of INF-γ, IL-6, and IL-17.

### IgG1 Autoantibodies on Disease Progression in MRL-*lpr/lpr* Mice

First, mouse IgG1 was isolated from the sera of highly IgG1-ANA-positive aged lupus-prone MRL-*lpr/lpr* mice (*n* = 20; age ≥ 15 weeks) (lupus IgG1) or age-matched control C57BL/6J mice (*n* = 20) (control IgG1) by immunoaffinity column chromatography using goat anti-mouse IgG1 (Thermo Fisher Scientific, Inc., San Jose, CA, USA) coupled to CNBr-activated Sepharose™ 4B (GE Healthcare, Chicago, USA) and quantified using the Pierce™ BCA Protein Assay Kit. The homogeneity and reactivity of the IgG1 antibody mixtures were analyzed by SDS-PAGE and IF staining with HEp-2 cells.

Second, the experimental design and time lines were followed as shown in **Figure 4A**. Briefly, eighteen 10-week-old female MRL*-lpr/lpr* mice were divided equally into three groups which were defined as the non-treated group, control IgG1 group (treated with IgG1 from age-matched control mice), and lupus IgG1 group (treated with IgG1 from aged MRL*-lpr/lpr* mice). Purified IgG1 was administered intravenously via the tail vein at 1.0 mg/(mouse∙week) for 10–18 weeks of age. Samples (blood, urine, spleen, and kidney) were collected at the indicated time points. All mice were sacrificed at 19 weeks of age for sample collection.

### Statistical Analysis

Statistical tests were performed using SPSS software version 23.0 (SPSS, Inc., Chicago, IL, USA) and GraphPad Prism software version 7.0 (GraphPad Software, Inc., San Diego, CA, USA). Two-group comparisons were performed using an independent-sample Student's *t* test, and multiple group comparisons were performed using one-way analysis of variance (ANOVA) followed by the Dunnett *post-hoc* test for normally distributed data. Non-parametric tests (Mann-Whitney U or Kruskal Wallis, as appropriate) were applied for non-normally distributed data. Differences with a *P*-value < 0.05 were considered to reflect a statistically significant difference. The data are expressed as the mean ± standard deviation (SD), counts, or percentage.

## Results

### Relationship Between Serum IgG4 Autoantibody Levels and Disease Activity in SLE Patients

**Figure 1:**
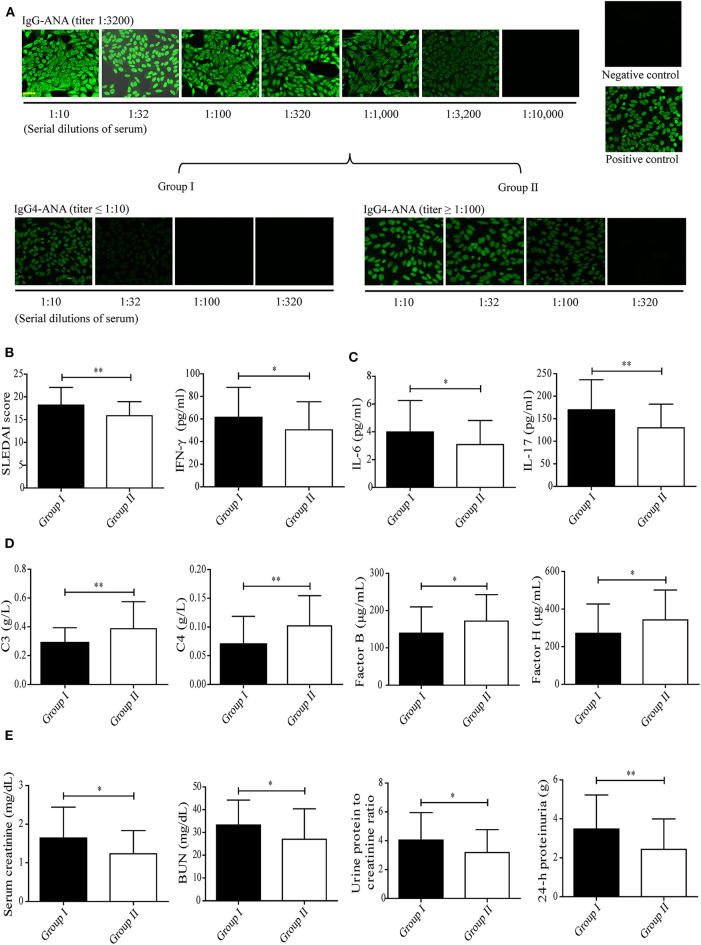
Correlation between serum levels of IgG4 autoantibody and disease activity in patients with SLE. **(A)** Serum levels of ANA-IgG (*n* = 106) and ANA-IgG4 in newly diagnosed patients in group I (*n* = 37) and group II (*n* = 32) were detected by IF staining of HEp-2 cells, and the fluorescence intensities were graded (scale bars, 50 μm). The SLEDAI score **(B)**, serum levels of inflammatory cytokines including IFN-γ, IL-6, and IL-17 **(C)**, serum levels of complement proteins including C3, C4, complement factor B, and complement factor H **(D)**, and serum creatinine, BUN, urine protein: creatinine, and 24-h proteinuria levels **(E)** in newly diagnosed patients with SLE of group I (*n* = 37) and group II (*n* = 32) were analyzed. *P < 0.05, **P < 0.01. The data **(B–E)** shown were analyzed via Student's *t*-test and are expressed as the mean ± SD. (ANA-IgG, anti-nuclear IgG antibody; ANA-IgG4, anti-nuclear IgG4 antibody; BUN, blood urea nitrogen).

To investigate whether IgG4 autoantibodies play a role in SLE, the levels of serum IgG4 autoantibodies and their correlation with disease activity (SLEDAI score) were evaluated in patients with SLE. We enrolled 106 newly diagnosed, untreated SLE patients with the same IgG autoantibody titer, as represented by IF staining of HEp-2 cells with anti-nuclear-IgG antibodies (ANA-IgG, 1:3,200) (Figure 1A). Group I was composed of 37 patients with lower serum IgG4 autoantibody titers as represented by anti-nuclear-IgG4 antibodies (ANA-IgG4, ≤ 1:10), and group II contained 32 patients with higher serum IgG4 autoantibody titers as represented by anti-nuclear-IgG4 antibodies (ANA-IgG4, ≥ 1:100), as shown in Figure 1A.

Much of the inflammation and tissue dysfunction mediated by autoantibody-autoantigen ICs in SLE occurs via activation of the complement system (4, 5, 7). We, therefore, investigated differences in inflammatory cytokine production and complement consumption in the two study groups. The results from the assessment of overall disease activity (as assessed by the SLEDAI score) was determined to be significantly lower in group II than in group I (Figure 1B, Table 1). In addition, inflammatory cytokine production (as assessed by the IFN-γ, IL-6, and IL-17 serum levels, Figure 1C) was significantly higher in group I than in group II. Complement consumption, which can be assessed by measuring the serum levels of C3, C4, and complement factors B and H of the alternative complement pathway (Figure 1D), were significantly higher in group I than in group II. In addition, renal function (as assessed by serum creatinine, BUN, urine protein: creatinine, and 24-h proteinuria levels) was significantly lower in group II than in group I (Figure 1E). No significant difference was found in anti-dsDNA IgG-positive rate and anti-nuclear IgG-positive rate in patients with SLE between group I than group II (Table 1).

These findings suggest that IgG4 autoantibodies likely attenuate SLE disease progression, perhaps by suppressing complement consumption and inflammatory cytokine production.

### IgG4 Autoantibodies Inhibit Complement Consumption by Autoantibody-Autoantigen ICs *in vitro*

**Figure 2:**
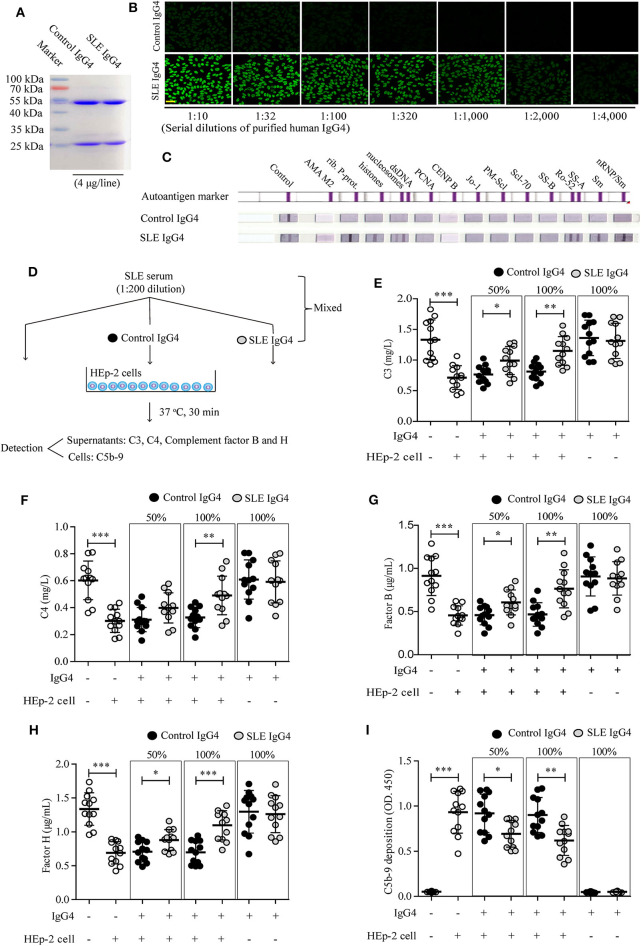
Analysis of purified human IgG4 and the effect of IgG4 on complement consumption by autoantibody-autoantigen ICs *in vitro*. Human IgG4 from the sera of healthy control subjects (*n* = 12) and newly diagnosed SLE patients (*n* = 12) was purified by immunoaffinity column chromatography and the antibody mixtures were analyzed by SDS-PAGE **(A)**, IF staining with HEp-2 cells (scale bars, 50 μm) **(B)**, and the ANA Euroline Profile 3 Kit **(C)**. **(D)** Schematic representation of the *in vitro* complement-consumption assay. Three independent experiments were performed. Consumption of complement proteins including C3 **(E)**, C4 **(F)**, complement factors B **(G)** and H **(H)**, as well as C5b-9 deposits **(I)** in cultured HEp-2 cells were detected after co-culturing HEp-2 cells with SLE serum, mixed with or without different concentrations of purified IgG4 (the IgG4: IgG ratio was 50 or 100%). As blank control, the first column of white circles from the left represents only sera from SLE patients (*n* = 12), without IgG4 and HEp-2 cells **(E–I)**; As positive control of the existence of autoantigens, the second column of white circles represents sera from SLE patients (*n* = 12), plus HEp-2 cells, but without IgG4 **(E–I)**. *P < 0.05, **P < 0.01, ***P < 0.001. The data **(E–I)** shown were analyzed via Student's *t*-tests and are presented as scatter plots and expressed as the mean ± SD.

Since complement consumption differed significantly between groups I and II, we next examined the role of IgG4 autoantibodies in complement consumption by autoantibody-autoantigen ICs *in vitro*. First, serum IgG4 was isolated from healthy control subjects and SLE patients by immunoaffinity-column chromatography. Next, the IgG4 antibody mixtures were analyzed by reducing gel SDS-PAGE (Figure 2A) and non-reducing gel SDS-PAGE (Supplemental Figure 1), IF staining with HEp-2 cells (Figure 2B), and the ANA Euroline Profile 3 Kit (Figure 2C). The results revealed high homogeneity of the purified SLE IgG4, which was present at high concentrations (titer 1:2000, 10 mg/mL) and showed broad reactivity against the major SLE autoantigens, including Smith (Sm), ribosomal P protein (Rib. P-prot.), dsDNA, nucleosomes, Sjögren's-syndrome-related antigen A (SS-A), and histones; however, no such reactivity was observed with control IgG4 (Figures 2A–C).

We then conducted *in vitro* testing to evaluate the role of IgG4 autoantibodies in complement consumption by autoantibody-autoantigen ICs at the cellular level (Figure 2D). The results revealed significantly higher C3 (Figure 2E), C4 (Figure 2F), C3a (Supplemental Figure 2A), and C5a (Supplemental Figure 2B) protein levels, complement factor B (Figure 2G) and H (Figure 2H) consumption, and C5b-9 deposition on HEp-2 cells co-cultured with SLE serum (Figure 2I). Alternatively, increased complement consumption and C5b-9 deposition were significantly inhibited by SLE IgG4 (in a concentration-dependent manner), however, not by control IgG4 (Figures 2E–I). In addition, both IgG4 isolated from healthy control subjects and SLE patients did not induce activation of the alternative pathway, as determined by no change of levels of complement factors B and H (key factors in the alternative pathway) when compared with control (Figures 2G,H).

These results demonstrate that IgG4 autoantibodies can inhibit complement consumption by autoantibody-autoantigen ICs *in vitro*.

### IgG4 Autoantibodies Inhibit Inflammatory Cytokine Production by SLE PBMCs *in vitro*

Next, since inflammatory cytokine production differed significantly between groups I and II, we also examined the role of IgG4 autoantibodies in inflammatory cytokine production by PBMCs *in vitro*.

**Figure 3:**
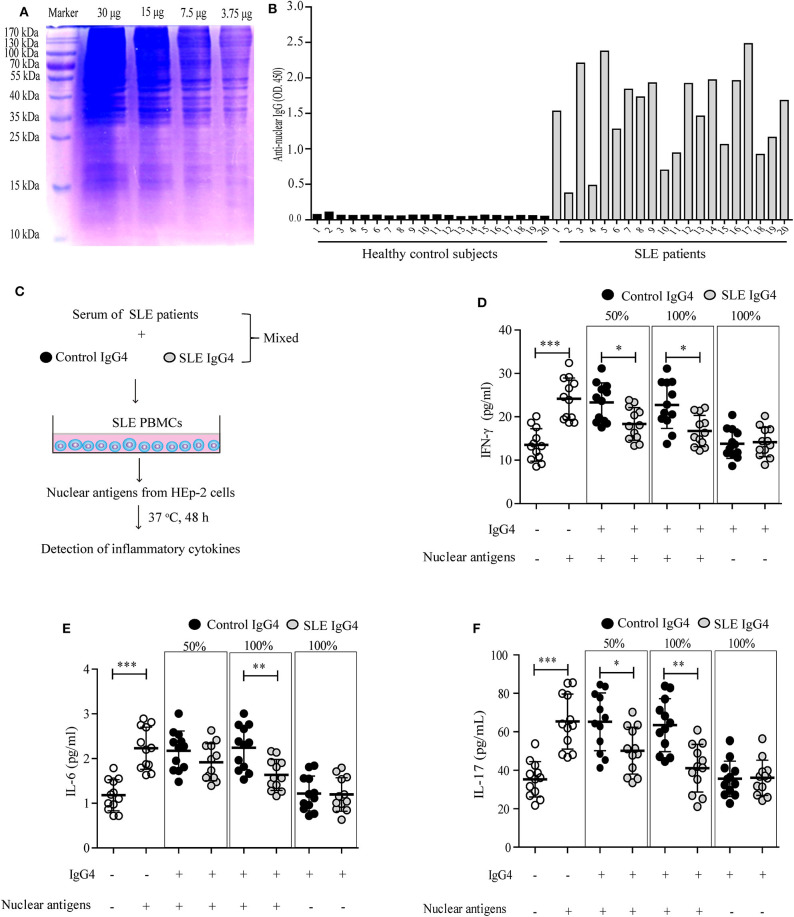
Analysis of purified IgG4 autoantigens and their effect on inflammatory cytokine production by SLE PBMCs *in vitro*. Purified nuclear autoantigens from HEp-2 cells were analyzed by SDS-PAGE **(A)**, and serum anti-nuclear IgG autoantibodies from healthy control subjects and SLE patients were detected by ELISA **(B)**. **(C)** Schematic representation of the *in vitro* inflammatory cytokine assays. Three independent experiments were performed. The production of inflammatory cytokines, including IFN-γ **(D)**, IL-6 **(E)**, and IL-17 **(F)** by autoantigen-stimulated SLE PBMCs were detected, mixed with or without different concentrations of purified IgG4 (the IgG4: IgG ratio was 50 or 100%). As blank control, the first column of white circles from the left represents only SLE PBMCs (*n* = 12, grown in DMEM/F12 supplemented with 25% serum of SLE patients), without IgG4 and nuclear antigens **(D–F)**; As positive control of the existence of nuclear antigens, the second column of white circles represents SLE PBMCs (*n* = 12, grown in DMEM/F12 supplemented with 25% serum of SLE patients), plus nuclear antigens, but without IgG4 **(D–F)**. *P < 0.05, **P < 0.01, ***P < 0.001. The data **(D–F)** shown were analyzed via Student's *t*-test and are presented as scatter plots and expressed as the mean ± SD.

First, autoantigens (represented by nuclear antigens), were isolated from HEp-2 cells and analyzed by SDS-PAGE (Figure 3A). Serum IgG autoantibodies from healthy control subjects and SLE patients were then detected by ELISA (Figure 3B). The prepared nuclear autoantigens included many different proteins that reacted well with IgG autoantibodies from SLE patients (Figures 3A,B). Second, an *in vitro* test was employed to evaluate the role of IgG4 autoantibodies in inflammatory cytokine production by SLE PBMCs (Figure 3C). IFN-γ (Figure 3D), IL-6 (Figure 3E), and IL-17 (Figure 3F) production by SLE PBMCs increased significantly following autoantigen activation. The increased production of these inflammatory cytokines was significantly inhibited by SLE IgG4 in a concentration-dependent manner, however, control IgG4 did not elicit this effect (Figures 3D–F). In addition, as baseline control, the amounts of cytokines in DMEM/F12 supplemented with 25% serum of highly ANA-IgG-positive SLE patients were IFN-γ (11.57 ± 3.24) pg/mL, IL-6 (0.86 ± 0.29) pg/mL, and IL-17 (32.41 ± 5.86) pg/mL.

These data indicate that IgG4 autoantibodies can inhibit inflammatory cytokine production by SLE PBMCs *in vitro*.

### IgG1 Autoantibodies Attenuate Disease Progression in Lupus-Prone MRL-*lpr/lpr* Mice

Our clinical data-correlation analysis and *in vitro* studies indicated that IgG4 autoantibodies can inhibit (i) complement consumption by autoantibody-autoantigen ICs and (ii) inflammatory cytokine production by SLE PBMCs. Followed, a lupus-prone MRL-*lpr/lpr* mouse model showed increased anti-nuclear antibody production, proteinuria, and kidney failure, etc. that reflects the pathology of human SLE (38) were used to further analyze the role of mouse IgG1 autoantibodies, which functionally resemble human IgG4 (14, 15, 39), in SLE.

**Figure 4:**
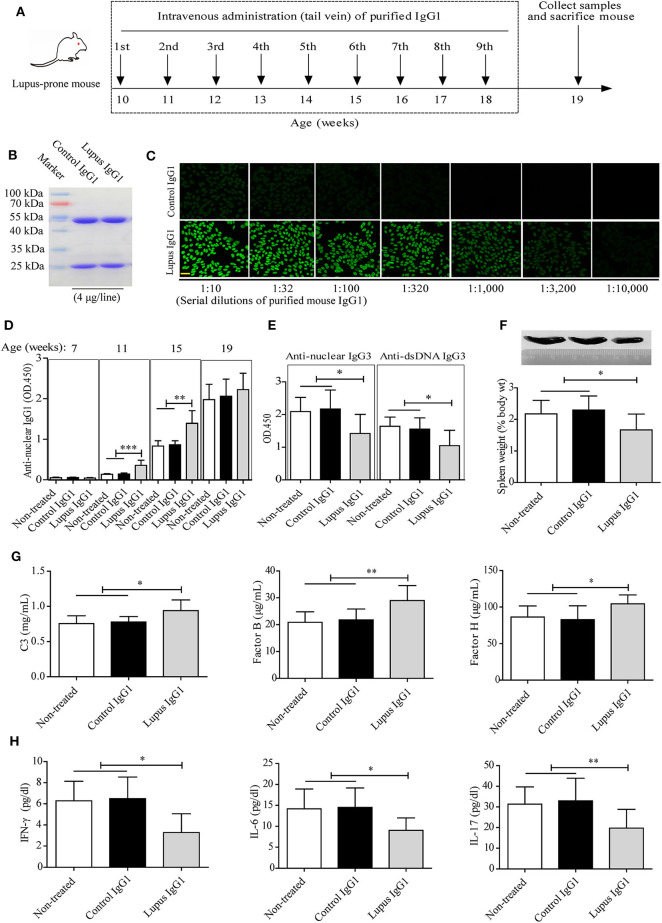
Effect of IgG1 autoantibodies on complement consumption and inflammatory cytokine production in MRL-*lpr/lpr* mice. **(A)** Experimental design and timeline. Serum mouse IgG1 from control mice (*n* = 20) and aged MRL-*lpr/lpr* mice (*n* = 20) was purified by immunoaffinity-column chromatography and the antibody mixtures were analyzed by SDS-PAGE **(B)** and IF staining with HEp-2 cells (scale bars, 50 μm) **(C)**. **(D,E)** Serum levels of autoantibodies in non-treated and control or lupus IgG1-treated MRL-*lpr/lpr* mice at 7, 11, 15, and 19 weeks of age (*n* = 6). **(F)** Representative images of the spleens of non-treated and control or lupus IgG1-treated MRL-*lpr/lpr* mice at 19 weeks of age (from left to right) and statistical analysis of the spleen weight as a percentage of the body weight (*n* = 6). **(G)** Serum levels of complement proteins including C3, complement factor B, and complement factor H, and **(H)** inflammatory cytokines including IFN-γ, IL-6, and IL-17 in non-treated and control or lupus IgG1-treated MRL-*lpr/lpr* mice at 19 weeks of age (*n* = 6). *P < 0.05, *P < 0.01, ***P < 0.001. The data **(D–G)** shown were analyzed via one-way ANOVA and are expressed as the mean ± SD.

We evaluated the effects of IgG1 injection on disease progression in MRL-*lpr/lpr* mice. To this end, serum IgG1 was isolated from control mice and aged MRL-*lpr/lpr* mice by immunoaffinity column chromatography, the IgG1 antibody mixtures were then analyzed by SDS-PAGE (Figure 4B) and IF staining with HEp-2 cells (Figure 4C). The results showed that the purified lupus IgG1 (not control IgG1) from aged MRL-*lpr/lpr* mice contained high IgG1 autoantibody concentrations (titer 1:3,200, 10 mg/mL) (Figure 4C).

Following the experimental design and timeline presented in Figure 4A, we found that the serum IgG1 autoantibody levels (represented by anti-nuclear IgG1) increased with age in MRL-*lpr/lpr* mice. Meanwhile, injecting lupus IgG1 at 10 weeks of age during the developing early-stage disease significantly increased anti-nuclear IgG1 levels in MRL-*lpr/lpr* mice at 11 and 15 weeks of age. However, no difference was found at 19 weeks of age, when compared with those of control IgG1-treated and non-treated groups (Figure 4D). Significant decreases in the serum anti-nuclear IgG3 and anti-dsDNA IgG3 levels (Figure 4E) as well as in the spleen weight: body weight ratio (Figure 4F) were also observed in lupus IgG1-injected MRL-*lpr/lpr* mice at 19 weeks of age, when compared with those of control IgG1-treated and non-treated groups.

In addition, lupus IgG1-injected MRL-*lpr/lpr* mice exhibited significantly higher serum C3 and complement factors B and H levels (Figure 4G), as well as significantly lower serum IFN-γ, IL-6, and IL-17 levels (Figure 4H) at 19 weeks of age, compared with those of control IgG1-treated and non-treated groups.

**Figure 5:**
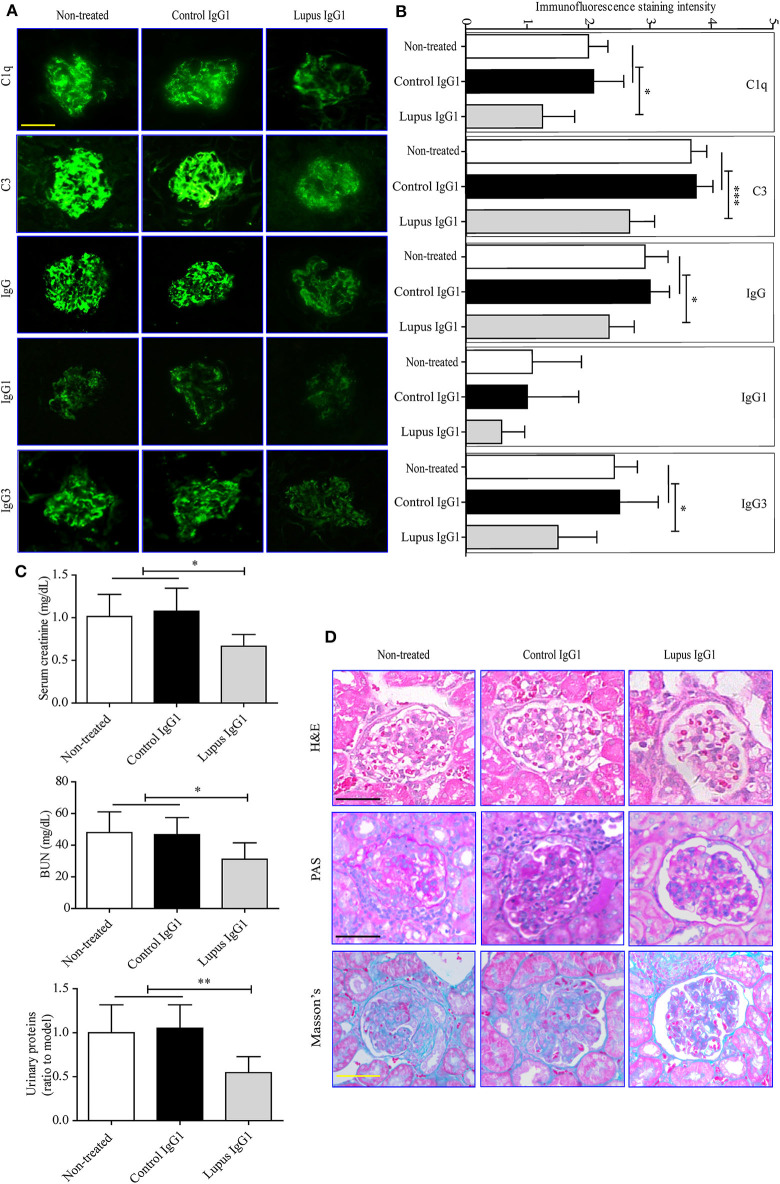
Effects of IgG1 autoantibodies on renal function and renal pathology in MRL-*lpr/lpr* mice. Representative fluorescence microscopy images **(A)** and statistical analysis **(B)** of C1q, C3, IgG1, and IgG3 deposition in the kidney of non-treated and control or lupus IgG1-treated MRL-*lpr/lpr* mice (*n* = 6) at 19 weeks of age (scale bars, 50 μm). Serum creatinine, BUN, and urinary proteins **(C)**, and light-microscopy examination of the histopathology of glomerulonephritis using H&E, PAS, and Masson's staining **(D)** in non-treated and control or lupus IgG1-treated MRL-*lpr/lpr* mice (*n* = 6) at 19 weeks of age (scale bars, 50 μm). *P < 0.05, **P < 0.01, ***P < 0.001. The data **(B,C)** shown were analyzed via one-way ANOVA and are expressed as the mean ± SD.

Finally, the effect of IgG1 injection on renal function in MRL-*lpr/lpr* mice was evaluated. The IF staining results indicated that lupus IgG1-injected MRL-*lpr/lpr* mice exhibited significantly less deposition of C1q, C3, IgG, IgG1, and IgG3 within their glomeruli at 19 weeks of age compared with that of control IgG1-treated and non-treated groups (Figures 5A,B). The serum creatinine, BUN, and urinary protein levels of lupus IgG1-injected MRL-*lpr/lpr* mice significantly decreased at 19 weeks of age compared with those of control IgG1-treated and non-treated group (Figure 5C). In addition, the renal histopathology of lupus IgG1-injected MRL-*lpr/lpr* mice was improved at 19 weeks of age compared with those of control IgG1-treated and non-treated groups (Figure 5D).

In addition, lupus IgG1, but not control IgG1, increased serum C3 and complement factors B levels (Supplemental Figure 3B), decreased serum IFN-γ and IL-17 levels (Supplemental Figure 3C), as well as decreased serum creatinine and BUN levels (Supplemental Figure 3D) in MRL-*lpr/lpr* mice by a dose-dependent manner within a range of 0.5 mg to 3.0 mg per mouse *in vivo*.

These findings suggest that IgG1 autoantibodies can attenuate disease progression in lupus-prone MRL-*lpr/lpr* mice *in vivo*.

## Discussion

This is a translational study for the development of novel biomedicine against SLE and other autoimmune diseases. Our findings demonstrated that IgG4 autoantibodies can attenuate disease progression in SLE likely by suppressing complement consumption and inflammatory cytokine production.

To determine whether IgG4 autoantibodies act as non-inflammatory molecules and exhibit a protective function in SLE due to their unique properties, we first enrolled newly diagnosed, untreated SLE patients with equal serum IgG autoantibody titer. These patients were then divided into group I and II according to their serum IgG4 autoantibody level. Next, we compared differences in disease activity-related markers (i.e., SLEDAI scores, serum levels of inflammatory cytokines, complement consumption, and kidney function) between two sub-groups of SLE patients. Interestingly, we found that patients in group II, who had higher (at least 10-fold) serum IgG4 autoantibody levels compared with those in group I, showed significantly lower levels of inflammation cytokine production, complement consumption, and SLEDAI scores. These findings indicate that IgG4 autoantibodies may play a protective role in SLE by suppressing complement consumption and inflammatory cytokine production. Similarly, a previous study reported that IgG4 autoantibodies against phospholipase A in beekeepers effectively inhibited complement activation by IgG1 autoantibodies in IgG1-containing ICs (40). These effects were determined to be induced primarily via inhibition of Clq binding to IgG1, which then reduced inflammation-induced injury (40). We also hypothesized that the half-molecule exchange promoted by Fc binding between two IgG4s conformationally altered IgG1, thereby optimizing the anti-inflammatory IgG4 response (41).

Low complement levels and cytokine activation are common in patients with SLE (4, 5). Thus, for our mechanistic study, we conducted *in vitro* experiments to investigate the effects of IgG4 autoantibodies on complement consumption and inflammatory cytokine production with samples from newly diagnosed SLE patients. The first key factor examined was the role of IgG4 autoantibodies in complement consumption by autoantibody-autoantigen ICs *in vitro*. Our results showed that C3 and C4 consumption, as well as C5b-9 deposition on HEp-2 cells co-cultured with SLE serum and HEp-2 cells, decreased significantly following treatment with IgG4 autoantibodies (but not control IgG4) in a concentration-dependent manner. It has been confirmed that complement abnormalities (4) and the function of complement molecules influence IC processing and transportation (7, 42). In addition, the ICs that can effectively activate complement molecules show greater pathological significance, with significantly more ICs found in SLE patients that were closely associated with SLE-related disease activities (43). It is well-recognized that IgG4 is incapable of activating the classical pathway (44, 45), although it is controversial whether it can activate the alternative pathway (44–46). Our findings demonstrate that both IgG4 from healthy control subjects and SLE patients did not activate the alternative pathway. In contrast, SLE IgG4 inhibited activation of the alternative pathway by other subclasses of autoreactive IgGs. These findings indicate that IgG4-autoantibodies can inhibit complement consumption by autoantibody-autoantigen ICs *in vitro*.

The second key factor investigated in our study was the effect of IgG4 autoantibodies on inflammatory cytokine production by SLE PBMCs. Our *in vitro* results showed that inflammatory cytokine production by SLE PBMCs increased significantly following autoantigen stimulation, which was subsequently inhibited by IgG4 autoantibodies in a concentration-dependent manner. Meanwhile the control IgG4 did not exhibit similar results. As reported, IgG4 is normally recognized as a non-inflammatory antibody, the primary role of which is to reduce (rather than accelerate) chronic immune activation (13). Above all, our *in vitro* findings demonstrate that IgG4 autoantibodies, not control IgG4, inhibit complement consumption by autoantibody-autoantigen ICs and inflammatory cytokine production by SLE PBMCs. Here, extraction kit used for nuclear antigens only isolates mainly soluble proteins, and the kits to isolate total nuclear extract (such as Sigma Nuclei Isolation Kit) will help us to isolates major lupus antigens including DNA and chromatin-bound proteins, etc. For an in-depth analysis of the role of IgG4 autoantibodies in SLE progression, we also conducted *in vivo* experiments with lupus-prone MRL-*lpr/lpr* mice. Our results show that lupus IgG1 injection could significantly increase serum anti-nuclear IgG1 levels in MRL-*lpr/lpr* mice through 19 weeks and that the increase in anti-nuclear IgG3 and anti-dsDNA IgG3 at 19 weeks of age were significantly inhibited in MRL-*lpr/lpr* mice treated with control IgG1 and non-treated groups. Consequently, the lupus IgG1-injected mice exhibited significant decreases in complement consumption and inflammatory cytokine production, as well as improved renal function and reduced renal histopathology. These findings demonstrate that IgG1-autoantibodies may suppress inflammatory responses and exert a protective function *in* MRL-*lpr/lpr* mice. Lilienthal *et al*. reported that murine IgG1 can inhibit the binding of murine IgG2a, IgG2b, and IgG3 to C1q *in vitro* (47). They also discussed that murine IgG1 and human IgG4 can potentially be used to block complement activation by sialylated IgG-subclass antibodies (47). Further, in a mouse model of cryoglobulinaemia, mouse IgG1 protected against renal disease (16), suggesting that Ig isotypes that poorly activate effector functions may be useful for inhibiting the immunopathology of IC-related disease (16). In addition, both control IgG1 and lupus IgG1 did not induce activation of the alternative pathway. Instead, lupus IgG1 inhibited activation of the alternative pathway by other subclasses of auto reactive IgGs, which is consistent with our findings in SLE patients.

Certain inherent limitations were noted within the current study. First, we noted that although the relative levels of autoantibodies prepared in this study, including human SLE IgG4 and mouse lupus IgG1, were assessed, few non-autoantibodies were also present in the autoantibody preparations. Therefore, we attempted to recover pure autoantibody solutions by including a second purification step, i.e., immunoaffinity-column chromatography using prepared autoantigens from HEp-2 cells (Figure 3A) coupled to CNBr-activated Sepharose™ 4B. IgG4 autoantibody production was very low and IgG4 activity was significantly decreased (data not shown), in part due to only a portion of the autoantigens being isolated or due to the coupling of only some autoantigens with sepharose. Hence, future studies will require successfully establishing a IgG1-deficient MRL-*lpr/lpr* mouse model to allow for more in-depth analysis of the potential pathogenic effects elicited by IgG1 autoantibody depletion in SLE, while taking into account the role that interference of non-autoreactive IgG1 depletion will have. Another limitation is that, for the *in vitro* study, the possibility of steric hindrance of a very high dose of IgG4 for antigen binding in the assays that may not be physiological.

**Figure 6:**
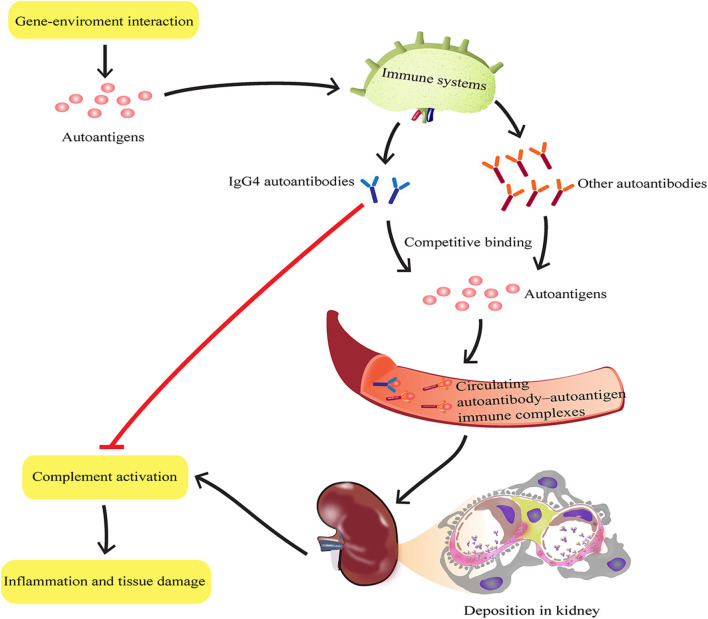
Schematic representation of IgG4 autoantibody-mediated attenuation of disease progression in SLE by suppressing complement consumption and inflammatory cytokine production. During SLE progression, the inflammatory response induced by each autoreactive IgG subtype depends on its ability to activate effector function. IgG4 autoantibodies may bind autoantigens completely with other subclasses of autoantibodies such as IgG1, IgG2, and IgG3, to form autoantibody-autoantigen ICs. The hinge region of IgG4 has a unique structure that determines its limited ability to activate effector function. Thus, compared with other subclasses of autoantibodies, IgG4 autoantibodies may attenuate disease progression in SLE by suppressing complement consumption and inflammatory cytokine production.

In conclusion, these findings, based on first hand materials (newly diagnosed SLE patients and isolated human PBMCs), also, with widely used classic spontaneous lupus-prone MRL-lpr/lpr mice, suggest that IgG4 autoantibodies attenuate SLE disease progression potentially by suppressing complement consumption and inflammatory cytokine production (Figure 6), which may provide novel therapeutic strategies against SLE and other autoimmune diseases.

## Data Availability Statement

All datasets generated for this study are included in the article/Supplementary Material.

## Ethics Statement

The studies involving human participants were reviewed and approved by the ethics committee of Affiliated Hospital of Guangdong Medical University. The patients/participants provided their written informed consent to participate in this study. The animal study was reviewed and approved by The Ethics Committee for Experimental Animals at Guangdong Medical University.

## Author Contributions

QP, HX, and HL designed the research and wrote the manuscript. LS, YH, JC, and JW performed the experiments and analyzed the data. AL, LY, and CY assisted in tissue sample collection and edited the manuscript. All authors read and approved the final manuscript.

### Conflict of Interest

The authors declare that the research was conducted in the absence of any commercial or financial relationships that could be construed as a potential conflict of interest.

## Supplementary Material

Supplemental Figure 1 The integrity of purified human IgG4 and mouse IgG1 were checked with SDS-PAGE (non-reducing). Briefly, 4.0 μg/line (human IgG4) and 2.0 μg/line (mouse IgG1) mixed with sample buffer was loaded onto the gels (3.9% stacking and 10% separating) for electrophoresis. SDS-PAGE (non-reducing) were performed and stained with coomassie brilliant blue R-250.

Supplemental Figure 2 Analysis of the effect of IgG4 on C3a and C5a consumption by autoantibody-autoantigen ICs *in vitro*. Consumption of complement C3a **(A)** and C5a **(B)** in cultured HEp-2 cells were detected after co-culturing HEp-2 cells with SLE serum, mixed with or without different concentrations of purified IgG4 (the IgG4: IgG ratio was 50 or 100%). As blank control, the first white bar from the left represents only sera from SLE patients (*n* = 12), without IgG4 and HEp-2 cells; As positive control of the existence of autoantigens, the second white bar represents sera from SLE patients (*n* = 12), plus HEp-2 cells, but without IgG4. ^*^*P* < 0.05, ^**^*P* < 0.01, ^***^*P* < 0.001. The data shown were analyzed via Student's *t*-tests and are expressed as the mean ± SD.

Supplemental Figure 3 Effect of different dose of IgG1 autoantibodies on complement consumption, inflammatory cytokine production, and renal function in MRL-*lpr/lpr* mice. **(A)** Experimental design and timeline. **(B)** Serum levels of complement proteins including C3 and complement factor B, and **(C)** inflammatory cytokines including IFN-γ and IL-17, and **(D)** serum creatinine and BUN in non-treated and control or lupus IgG1-treated MRL-*lpr/lpr* mice at 19 weeks of age (*n* = 6). ^*^*P* < 0.05, ^**^*P* < 0.01, ^***^*P* < 0.001. The data **(B–D)** shown were analyzed via one-way ANOVA and are expressed as the mean ± SD.
