# Supplementary figures and images for: IgG4 Autoantibodies Attenuate Systemic Lupus Erythematosus Progression by Suppressing Complement Consumption and Inflammatory Cytokine Production
Source: Front Immunol. 2020 Jun 17;11:1047. doi: 10.3389/fimmu.2020.01047 (PMC7311789; doi:10.3389/fimmu.2020.01047)

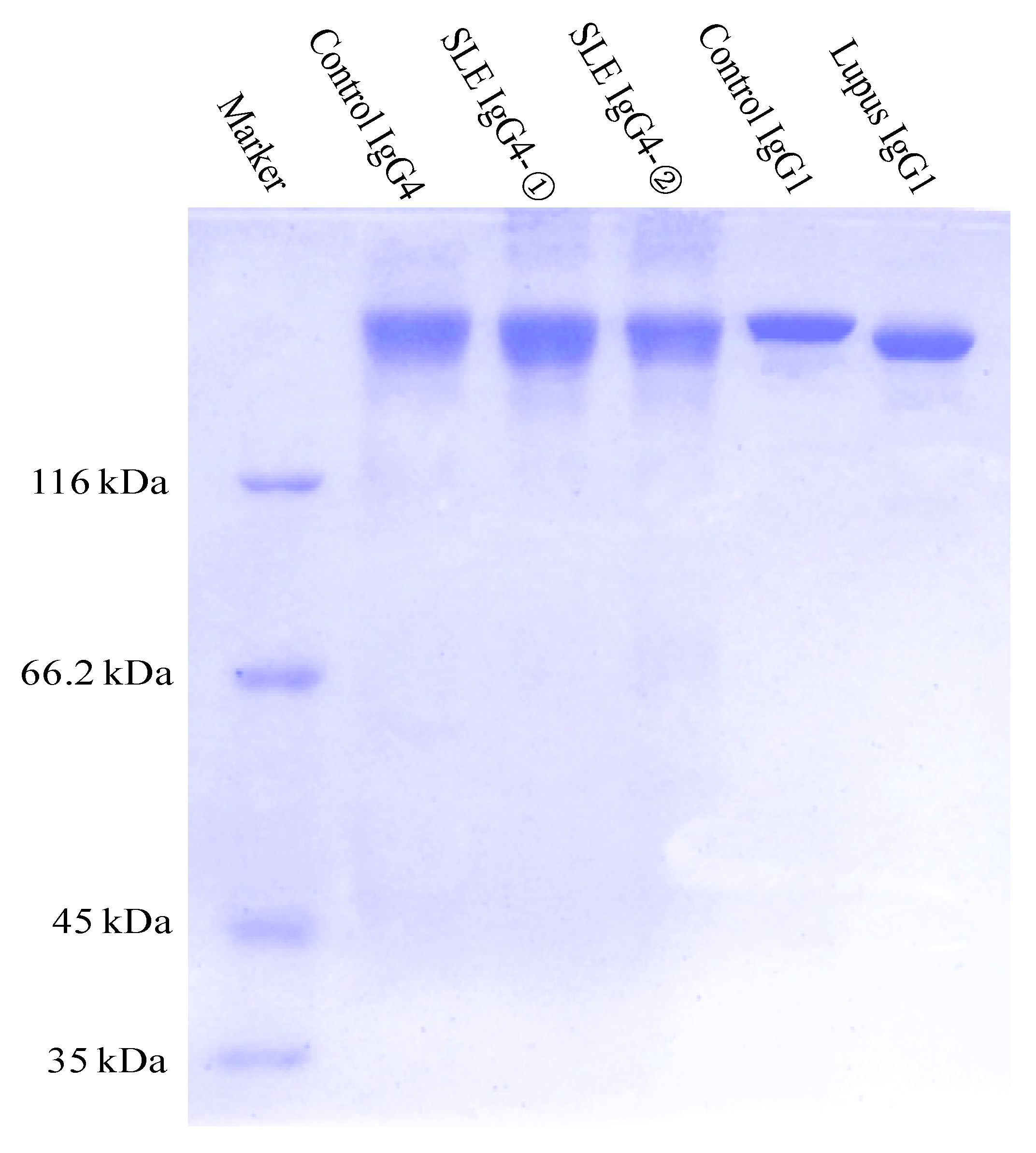

Supplement: Supplemental Figure 1 — The integrity of purified human IgG4 and mouse IgG1 were checked with SDS-PAGE (non-reducing). Briefly, 4.0 μg/line (human IgG4) and 2.0 μg/line (mouse IgG1) mixed with sample buffer was loaded onto the gels (3.9% stacking and 10% separating) for electrophoresis. SDS-PAGE (non-reducing) were performed and stained with coomassie brilliant blue R-250. [file Image_1.tiff]

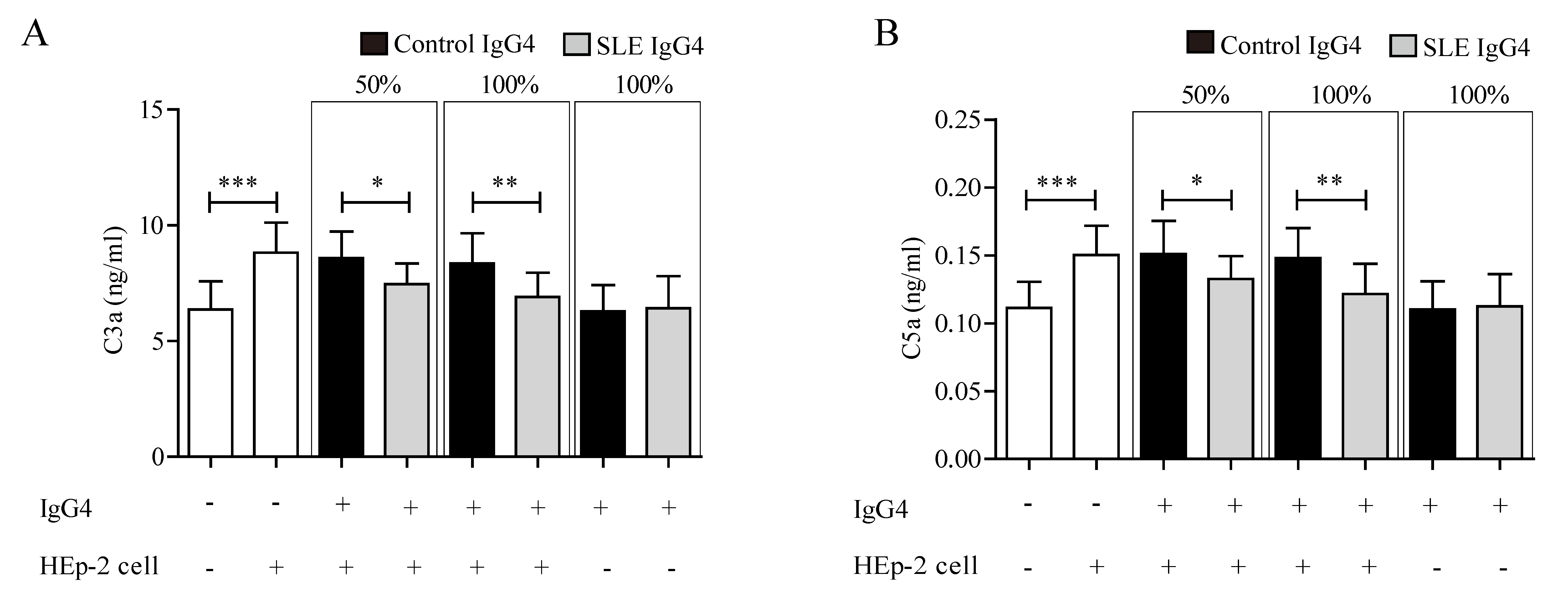

Supplement: Supplemental Figure 2 — Analysis of the effect of IgG4 on C3a and C5a consumption by autoantibody-autoantigen ICs in vitro. Consumption of complement C3a (A) and C5a (B) in cultured HEp-2 cells were detected after co-culturing HEp-2 cells with SLE serum, mixed with or without different concentrations of purified IgG4 (the IgG4: IgG ratio was 50 or 100%). As blank control, the first white bar from the left represents only sera from SLE patients (n = 12), without IgG4 and HEp-2 cells; As positive control of the existence of autoantigens, the second white bar represents sera from SLE patients (n = 12), plus HEp-2 cells, but without IgG4. *P < 0.05, **P < 0.01, ***P < 0.001. The data shown were analyzed via Student's t-tests and are expressed as the mean ± SD. [file Image_2.tiff]

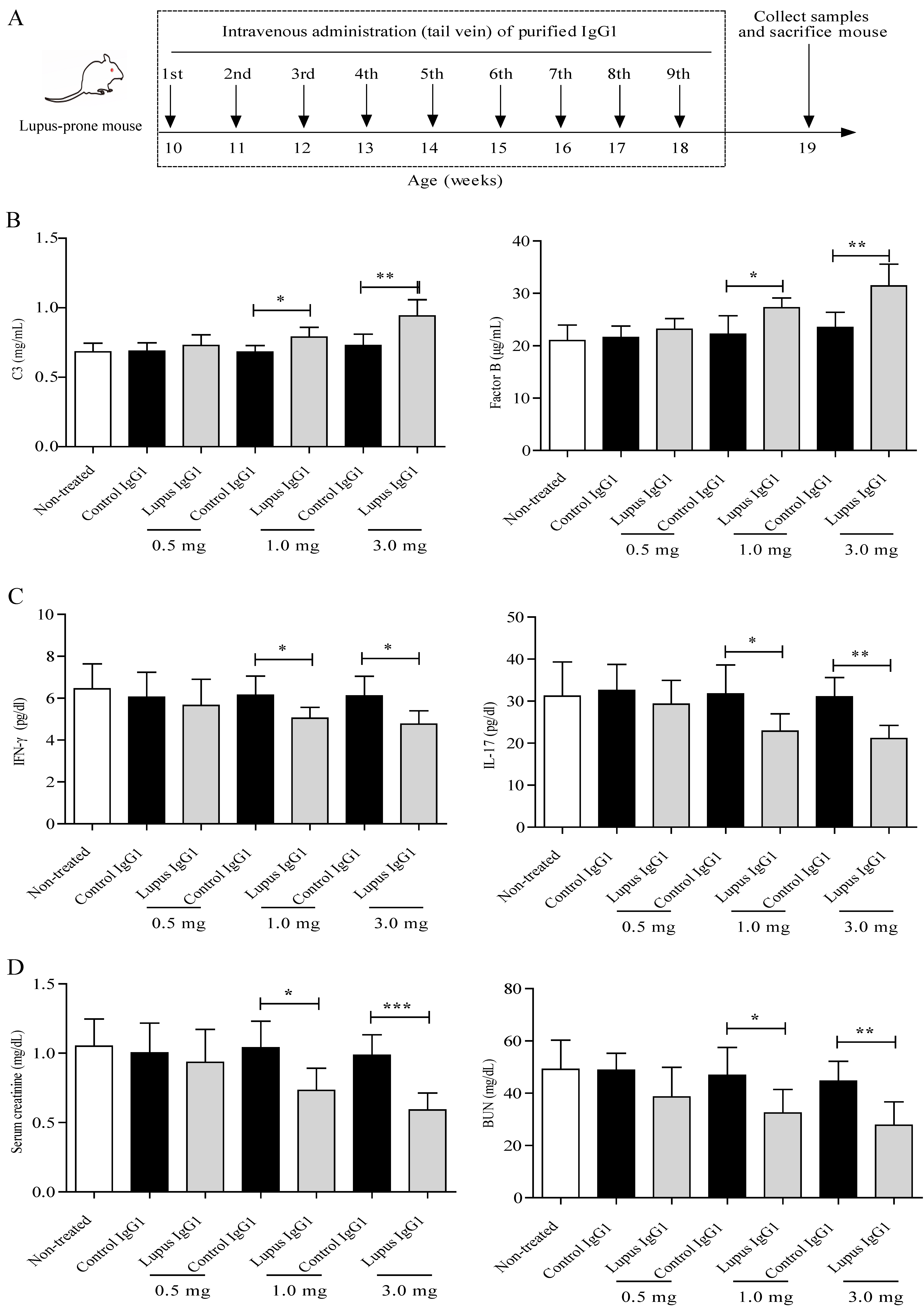

Supplement: Supplemental Figure 3 — Effect of different dose of IgG1 autoantibodies on complement consumption, inflammatory cytokine production, and renal function in MRL-lpr/lpr mice. (A) Experimental design and timeline. (B) Serum levels of complement proteins including C3 and complement factor B, and (C) inflammatory cytokines including IFN-γ and IL-17, and (D) serum creatinine and BUN in non-treated and control or lupus IgG1-treated MRL-lpr/lpr mice at 19 weeks of age (n = 6). *P < 0.05, **P < 0.01, ***P < 0.001. The data (B–D) shown were analyzed via one-way ANOVA and are expressed as the mean ± SD. [file Image_3.tiff]
